# Characterization and Functional Analysis of the *FBN* Gene Family in Cotton: Insights into Fiber Development

**DOI:** 10.3390/biology14081012

**Published:** 2025-08-07

**Authors:** Sunhui Yan, Liyong Hou, Liping Zhu, Zhen Feng, Guanghui Xiao, Libei Li

**Affiliations:** 1 College of Advanced Agriculture Sciences, Zhejiang A&F University, Lin’an, Hangzhou 311300, China; 202420020118@stu.zafu.edu.cn (S.Y.); fengzhen@zafu.edu.cn (Z.F.); 2 College of Life Sciences, Shaanxi Normal University, Xi’an 710119, China; hlya0504@163.com; 3 National Key Laboratory of Cotton Bio-Breeding and Integrated Utilization, School of Life Science, Henan University, Kaifeng 475004, China; zhuliping0903@163.com

**Keywords:** cotton, *FBN* gene family, fiber development, hormone response, gene expression

## Abstract

Fibrillins (FBNs) are indispensable for plant growth and development, orchestrating multiple physiological processes. However, the precise functional role of *FBNs* in cotton fiber development remains uncharacterized. This study reports a genome-wide characterization of the *FBN* gene family in cotton. A total of 28 *GhFBN* genes were identified in upland cotton, with systematic analyses of their phylogenetic relationships, protein motifs, gene structures, and hormone-responsive *cis*-regulatory elements. Expression profiling of *GhFBN1A* during fiber development revealed stage-specific activity across the developmental continuum. Transcriptomic analyses following hormone treatments demonstrated upregulation of *GhFBN* family members, implicating their involvement in hormone-mediated regulatory networks governing fiber cell development. Collectively, this work presents a detailed molecular characterization of cotton *GhFBN*s and establishes a theoretical foundation for exploring their potential applications in cotton breeding programs aimed at improving fiber quality.

## Simple Summary

The FBN gene family in cotton was characterized in this study: 28 members were identified in upland cotton (*Gossypium hirsutum*), with comparable numbers in other cotton species. Allotetraploid species harbor approximately twice as many FBN genes as diploids, indicating lineage-specific expansion during polyploidization. Phylogenetic and structural analyses showed these genes cluster into 11 groups, share conserved motifs and the PAP-fibrillin domain, and contain hormone-responsive cis-elements. Expression profiling revealed that specific *GhFBN* genes, such as *GhFBN2-2*, are highly expressed during key fiber development stages. Hormone treatments demonstrated that *GhFBN1A-2* is induced by GA_3_, which promotes fiber growth, but repressed by MeJA, while *GhFBN11-2* is downregulated by GA_3_ and IAA. These findings highlight *FBN*s’ role in hormone-mediated cotton fiber development, providing targets for improving fiber quality in breeding programs.

## 1. Introduction

Fibrillin, a plastid lipid-associated protein (PAP) first identified in red rose (*Rosa rugosa*) and bell pepper (*Capsicum annuum*), is conserved across photosynthetic organisms spanning from cyanobacteria to higher plants [1,2,3,4,5,6,7]. Since its discovery, extensive studies have shown that fibrillin is remarkably conserved across a wide spectrum of photosynthetic organisms, ranging from simple cyanobacteria to complex higher plants. This conservation implies that fibrillin plays fundamental roles in photosynthetic processes that are essential for the survival and growth of these organisms. For example, in cyanobacteria, fibrillin proteins might be involved in maintaining the stability of the photosynthetic apparatus, which is crucial for their autotrophic lifestyle [8]. Fibrillins, initially characterized by their subcellular localization and functional specializations in distinct organelles, have been variously denominated as plastid lipid-associated protein (PAP), plastoglobule protein (PGL), chloroplast drought-induced stress protein of 34 kDa (CDSP34), and chromoplast-specific carotenoid-associated protein (ChrC) [2,3,9,10].

Research on the subcellular localization of FBN proteins is crucial for understanding their functions. Multiple studies have shown that FBN proteins are predominantly located in chloroplasts, distributed across the stroma, thylakoids, and plastoglobules (PGs) [6,11,12]. FBN proteins are also present in plastids other than chloroplasts, with relevant studies having elucidated their distribution in plastid-containing organisms and the significance of their presence in non-chloroplast plastids [4,13]. Overall, FBN proteins’ subcellular localization provides a spatial basis for their involvement in photosynthesis, lipid metabolism, plant development, and stress responses.

Several investigations have shown that FBNs centrally regulate key physiological processes in diverse plant species, including photosynthetic mechanisms, stress response, and plant growth and development [14,15,16,17]. Among the 14 FBN isoforms in *Arabidopsis*, FBN1a, FBN1b, and FBN2 regulate jasmonic acid biosynthesis under light or low-temperature stress [12,18]. In bell pepper, FBN1 protein levels correlate positively with fruit ripening, while in potato, high expression of C40.4 (FBN1) enhances tuber carotenoid content [19]. The *OsFBN* gene in rice is induced by extreme temperature stress [10,20]. In diverse plant species, including cucumber (*Cucumis sativus*), tomato (*Solanum lycopersicum*), sweet orange (*Citrus sinensis*) and mustard (*Brassica juncea*), the expression of FBN proteins has been reported [21,22,23,24].

Cotton, as the world’s premier natural fiber crop, serves as an ideal model system for studying cell elongation [25,26]. *Gossypium hirsutum*, domesticated approximately 8000 years ago, has become the predominant cultivated cotton species due to its production of relatively long fiber cells that are well-adapted to modern textile manufacturing processes [25]. The fiber quality of cotton directly affects the economic efficiency of the textile industry. Cotton fiber cell development is typically divided into five overlapping stages: cell initiation, elongation, transitional wall thickening, secondary cell wall thickening, and maturation [27]. The elongation stage is a critical period that determines fiber length, an important trait that directly influences the economic value of cotton [28]. Despite the established role of FBNs in plant development across multiple species, their function in cotton fiber development remains uncharacterized. Here, we identified the *GhFBN* gene family from four cotton species’ genomic data via bioinformatics, characterizing their structures, chromosomal locations, phylogeny, collinearity, and expression profiles to reveal functional diversity. This study deepens the understanding of cotton *GhFBN* genes, highlights their potential in enhancing fiber quality, and provides valuable candidate genes and theoretical support for molecular breeding to improve cotton fiber traits in agricultural practice.

## 2. Materials and Methods

### 2.1. Identification of Cotton GhFBN Genes

Amino acid sequences of the *Arabidopsis FBN* gene family were obtained from the *Arabidopsis* Information Resource (https://www.arabidopsis.org, (accessed on 5 April 2025)). Subsequently, these sequences were used as query sets to conduct local BLASTP searches against the reference genome assemblies of *Gossypium hirsutum*, *Gossypium raimondii*, *Gossypium arboreum* and *Gossypium barbadense* (https://yanglab.hzau.edu.cn/CottonMD, (accessed on 5 April 2025)), with a significance criterion of E-value < 1 × 10^−5^. The hidden Markov model (HMM) profile for the FBN domain (Pfam accession: PF04755) was retrieved from the Pfam database [29]. This profile was subsequently employed to screen protein databases using HMMER v3.0 (http://hmmer.org/, (accessed on 20 April 2025)), with a stringent E-value cutoff of <0.01 to ensure the reliability of the search results. The identities of the screened proteins were further verified by querying the SMART [30] and InterPro [31] databases. The identified cotton *FBN* genes were designated based on their sequence homology with *Arabidopsis* orthologs.

### 2.2. Multiple Alignment and Phylogenetic Analysis

Multiple sequence alignments and phylogenetic analyses were performed using MEGA 11.0 software. The Neighbor Joining (NJ) method was employed for phylogenetic tree reconstruction, with bootstrap support values calculated based on 1000 replicates to assess nodal confidence. All other algorithmic parameters were maintained at their default settings.

### 2.3. Structure Analysis and Cis-Element Prediction

Motif analysis was conducted using the MEME Suite v5.5.8 [32]. Promoter regions (2000 bp upstream of the translation start site) were extracted, and cis-regulatory elements were predicted using PlantCARE (https://bioinformatics.psb.ugent.be/webtools/plantcare/html/, (accessed on 10 May 2025)). The gene structure and motif analysis results were visualized using TBtools software v2.225 [33].

### 2.4. Chromosomal Location and Collinearity Analysis

Chromosomal location information for genes was extracted from the genome GFF3 annotation data, and TBtools v2.225 software was employed to generate chromosomal mapping diagrams and conduct gene collinearity analysis [33].

### 2.5. In Vitro Ovule Culture and RNA-Seq

Ovules were harvested from +1 DPA upland cotton flowers. Following surface sterilization with 75% (*w*/*v*) ethanol, the ovules were dissected under sterile conditions and transferred to ovule in vitro culture medium [34]. For the experimental groups, culture media were prepared with final concentrations of 0.1 mM MeJA (methyl jasmonate), 0.5 mM GA_3_ (gibberellin A_3_), or 5 mM IAA (indole-3-acetic acid). All hormones were purchased from Beijing Solarbio Science & Technology Co., Ltd. (Beijing, China). Following independent cultivation at 30 °C for 10 days, total RNA was extracted from the collected samples, with 2 μg of RNA used for RNA-seq library construction. Transcriptome sequencing (RNA-seq) was performed on the Illumina NovaSeq 6000 platform, and subsequent data analysis was carried out by Wuhan IGENEBOOK Biotechnology Co. (Wuhan, China). Three independent biological replicates were included in the experiment.

### 2.6. Evolutionary Selection Pressure Analysis

Using the Easy Ka/Ks Calculator in TBtools v2.225 software [33], we calculated the nonsynonymous (Ka) and synonymous (Ks) substitution rates for duplicated *FBN* gene pairs that had been aligned by MEGA 11.0 software. According to the Ka/Ks ratio criteria (Ka/Ks > 1 indicating positive selection, Ka/Ks = 1 indicating neutral selection, and Ka/Ks < 1 indicating purifying selection [35]), we evaluated the selection pressure patterns for each gene pair.

### 2.7. Expression Pattern Analysis of Cotton FBN Genes

Transcriptome datasets from diverse tissues (ovules at 0 days post-anthesis (DPA), and fibers at 5, 10, 15, 20, and 25 DPA) [36] and hormone conditions (MeJA, GA_3_ and IAA treatments) were utilized. Gene expression levels were estimated as fragments per kilobase of exon per million reads (FPKM) using Cufflinks, normalized, and visualized via heatmaps generated with TBtools software [33].

### 2.8. qRT-PCR Analysis

Total RNA was extracted separately from ovules at 0 DPA and developing fiber tissues at 5, 10, 15, and 20 DPA of Jin668 using the FastPure Universal Plant Total RNA Isolation Kit (Vazyme Biotechnology, Nanjing, China). First-strand cDNA was synthesized from 1 μg of total RNA using the HiScript II Q RT SuperMix for qPCR (Vazyme Biotechnology) according to the manufacturer’s protocol. Quantitative real-time PCR (qRT-PCR) was performed using the ChamQ SYBR qPCR Master Mix (Vazyme Biotechnology), with three biological replicates per gene. The cotton ubiquitin gene UBQ7 (GenBank accession: AY189972) was used as an internal reference, and primer sequences are listed in Table S1. Relative gene expression levels were calculated using the 2^−ΔΔCT^ method [37].

## 3. Results

### 3.1. Identification of FBN Genes in Cotton

To identify FBN genes in cotton, we used 14 *Arabidopsis* FBN amino acid sequences as queries for BLASTP and HMMER3.0 searches, and identified 28 FBN proteins in *G. hirsutum* (Gh), 28 in *G. barbadense* (Gb), 13 in *G. arboreum* (Ga) and 15 in *G. raimondii* (Gr) (Table 1). Comparative genomic analysis revealed that allopolyploid cotton species (*G. hirsutum* and *G. barbadense*) possessed approximately twice the *FBN* gene count of diploid *G. arboreum* and *G. raimondii* (Table 1), indicating lineage-specific expansion of the *FBN* gene family during cotton polyploidization. In *G. arboreum*, the lengths of *FBN* genes ranged from 1031 bp to 5543 bp, encoding polypeptides composed of 215 to 677 amino acids. The predicted molecular weights spanned from 24.35 kDa to 76.67 kDa, with theoretical isoelectric points varying between 4.54 and 9.86 (Table 1). In *G. raimondii*, *FBN* gene lengths varied between 728 bp and 6103 bp, encoding polypeptides composed of 423 to 2031 amino acids. Predicted molecular weights spanned 15.87 kDa to 76.79 kDa, with theoretical isoelectric points ranging from 4.49 to 9.76 (Table 1). Similarly, in *G. barbadense* and *G. hirsutum*, notable variations were observed in the *FBN* gene attributes. Their gene lengths ranged from 609 bp to 12,587 bp, isoelectric points from 4.4 to 9.8, and molecular weights from 12.79 kDa to 76.68 kDa (Table 1). These findings suggest that the *FBN* gene family in cotton has undergone evolutionary expansion.

### 3.2. Phylogenetic Relationships of FBN Genes

**Figure 1:**
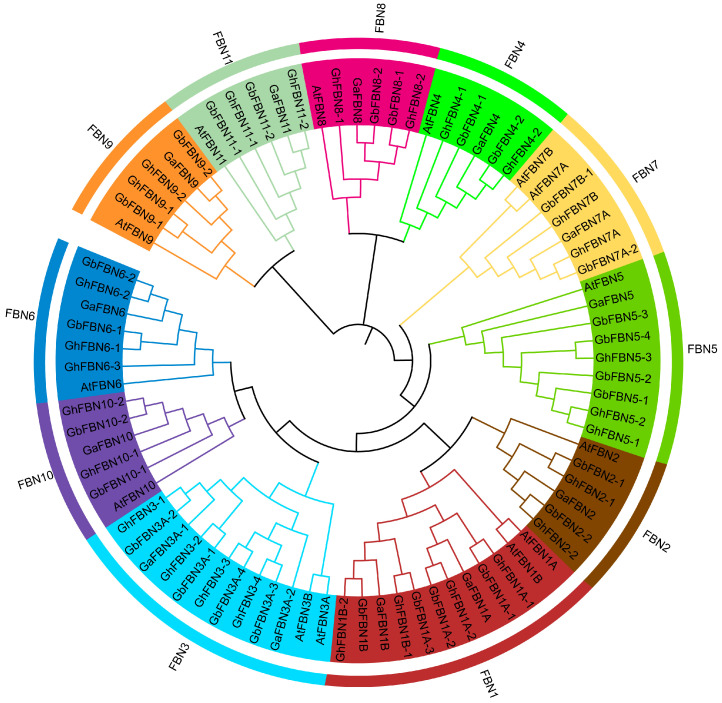
The phylogenetic tree of the FBN family in cotton. Phylogenetic analysis, performed using MEGA11.0, included *Arabidopsis* (14), *G. hirsutum* (28), *G. barbadense* (28), *G. arboreum* (13) and *G. raimondii* (15) sequences. Different subfamilies are color-coded for clear differentiation.

To clarify the evolutionary relationships of the *FBN* gene family in cotton, we constructed a phylogenetic tree using the Neighbor Joining (NJ) method with the FBN protein sequences from *A. thaliana*, *G. arboreum*, *G. raimondii*, *G. hirsutum* and *G. barbadense*. Cotton *FBN* genes from *G. arboreum*, *G. raimondii*, *G. hirsutum* and *G. barbadense* are named by their phylogenetic positions relative to *AtFBN* genes. Phylogenetic analysis revealed that FBNs are classified into 11 groups (Figure 1). The groups FBN2, FBN4, FBN8, FBN9, FBN10, and FBN11 exhibited a highly conserved evolutionary pattern. These gene groups experienced independent expansions in *G. arboreum*, *G. raimondii*, *G. hirsutum*, and *G. barbadense*, and manifested a one-to-one orthologous relationship. During evolution in *G. arboreum*, the genes *GaFBN3B* and *GaFBN7B* were lost, whereas *G. raimondii* underwent losses of *GrFBN1B*, *GrFBN3B*, and *GrFBN7A*. The hybridization events in allotetraploid cotton species (*G. hirsutum* and *G. barbadense*) might have driven this unbalanced evolutionary trajectory.

### 3.3. Structure Analysis of Cotton GhFBN Genes

**Figure 2:**
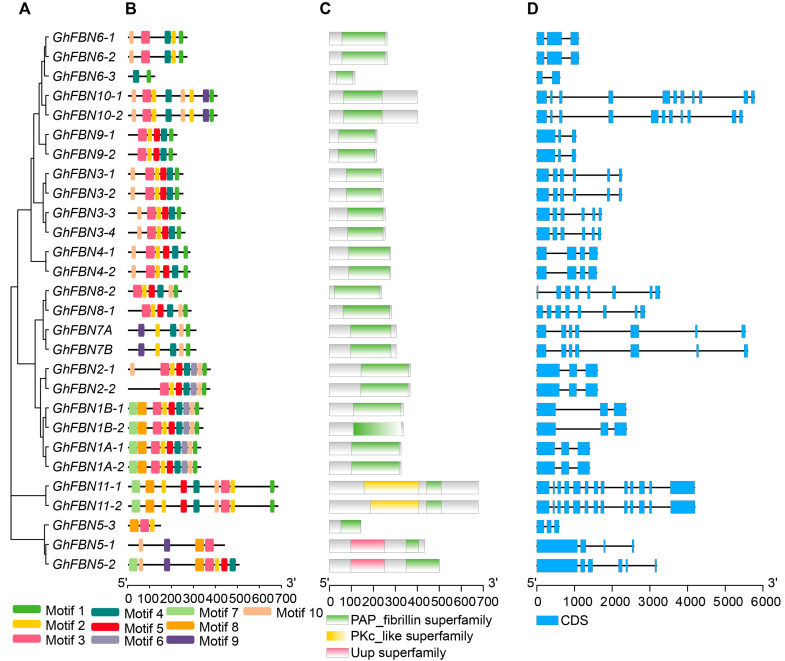
The structural distribution patterns of the 28 *GhFBN* genes. (**A**) Construction of a phylogenetic tree using GhFBN protein sequences. (**B**) The distribution of MEME conserved motifs, where different conserved motifs are indicated by colored boxes. (**C**) The distribution of the PAP fibrillin conserved domain, with the green square signifying this conserved domain. (**D**) The gene structure of *GhFBN*, in which exons are represented by blue boxes.

To comprehensively explore the structural diversity of *FBN* genes, we conducted an analysis of the sequence structures, motifs, and conserved domains of the identified *FBN* genes in *G. hirsutum*, guided by phylogenetic analysis (Figure 2A). With the exceptions of GhFBN6-3 and GhFBN5-1, the remaining genes shared similar motifs (Figure 2B). All members of the FBN family possess the PAP-fibrillin domain, a characteristic structural feature that defines this gene family (Figure 2C). Moreover, the exon/intron structures of *FBN* genes within the same group were strikingly alike compared to those in other groups (Figure 2D). These findings imply that genes with unique structural features might perform specialized biological functions.

### 3.4. Chromosomal Location and Gene Duplication of GhFBN Genes

**Figure 3:**
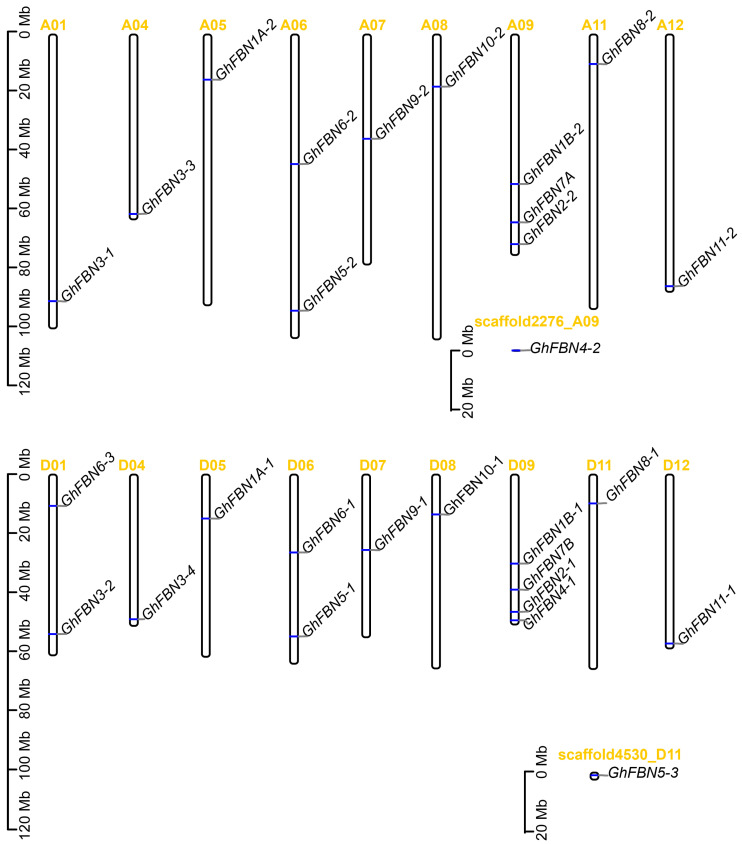
The chromosomal locations of *FBN* in the Gossypium hirsutum species. Gene IDs are annotated on the right, whereas vertical ideograms on the left depict both gene loci and chromosomal lengths. Mb, megabase.

**Figure 4:**
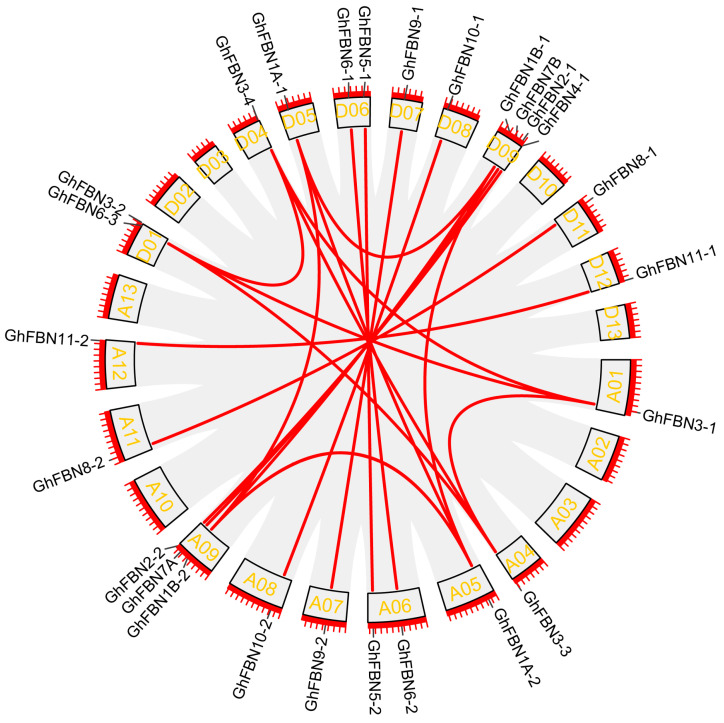
The distribution and duplication of *GhFBN* genes in *G. hirsutum*. The distribution of *GhFBN* genes across the 24 chromosomes of the *G. hirsutum* genome. Duplicated genes are connected by red lines.

**Figure 5:**
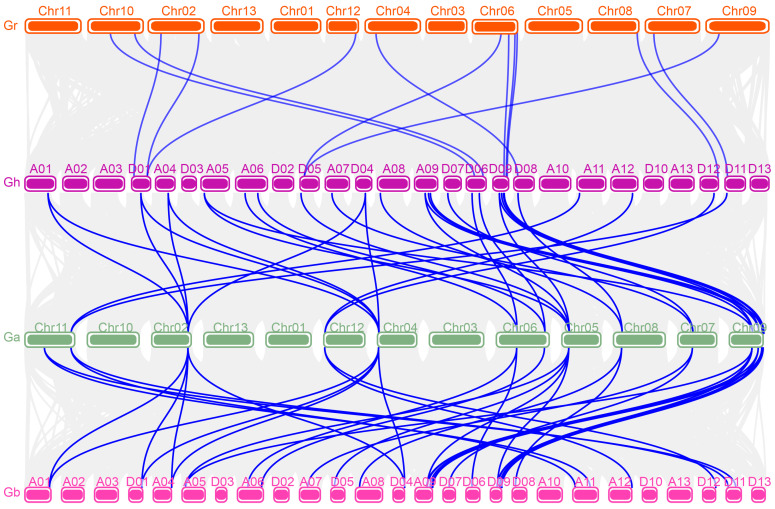
The collinearity of *FBN* genes among various cotton species. The chromosomes of *G. raimondii*, *G. hirsutum*, *G. arboreum* and *G. barbadense* are shown in different colors, marked in orange, purple, green, and pink, respectively. The blue lines indicate the collinear relationships among *FBN* genes.

To investigate the chromosomal distribution of *GhFBN* genes, each *GhFBN* gene was mapped to its corresponding chromosome using gene information from the NBI database. For a deeper exploration of *GhFBN* gene evolution across four cotton species, genome duplication events including whole-genome duplication (WGD), segmental duplication, and tandem duplication were examined. In *G. hirsutum*, 28 *GhFBN* genes display a heterogeneous distribution across the 18 chromosomes. Specifically, there are 13 *GhFBN* genes in the A chromosome group and 15 *GhFBN* genes in the D chromosome group (Figure 3). Chromosome D09 harbors the highest number of *GhFBN* genes, with four. In contrast, *GhFBN4-2* and *GhFBN5-3* are mapped to scaffolds. To decipher the expansion pattern of the *GhFBN* gene family in *G. hirsutum*, Circos analysis was carried out. The results demonstrate that 26 *GhFBN* family genes have experienced tandem duplication and are dispersed across chromosomes A01, A04, A05, A06, A07, A08, A09, A11, D01, D04, D05, D06, D07, D08, D09 and D11 (Figure 4). The remaining two genes are scattered within the *G. hirsutum* genome. We further delved into the *FBN* gene family in *G. hirsutum*, *G. arboreum*, *G. raimondii* and *G. barbadense*, and explored the gene duplication patterns of *FBN* genes in *G. arboreum* and *G. barbadense* (Figure 5). The duplicate gene pairs of these four cotton species reveal the basis for polyploidization and large-scale expansion of the *FBN* gene family during the evolutionary process.

### 3.5. Selection Pressure Ka/Ks Analysis

To elucidate the selective pressures acting on *GhFBN* genes during gene duplication in upland cotton, this study comprehensively analyzed coding sequences of all intragenomic paralogous pairs. Homologous coding regions free of frameshift mutations were identified through sequence alignment, followed by computation of nonsynonymous (Ka) and synonymous (Ks) substitution rates for each paralog pair using TBtools. Evolutionary selective pressures were inferred based on Ka/Ks ratio analyses. In *G. hirsutum GhFBNs*, 17 gene pairs exhibited Ka/Ks < 0.5 and 3 displayed ratios of 0.5–1, with strong purifying selection and high sequence conservation inferred in the majority and near-neutral evolution detected in the remainder (Table 2). These findings suggest that functional divergence was constrained by dosage effects or evolutionary time constraints post-whole-genome/segmental duplication.

### 3.6. Analysis of Putative Cis-Acting Elements in GhFBN Promoters

**Figure 6:**
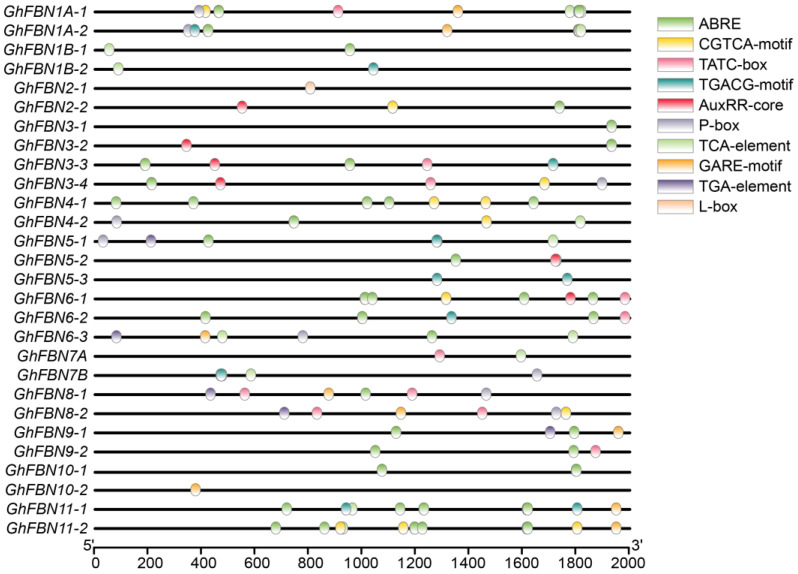
Analysis of *cis*-acting elements in the upstream region of the *GhFBN* gene promoter. Boxes in different colors represent the uniquely identified *cis*-acting elements.

To further explore the biological activities of *GhFBNs* in *G. hirsutum*, we obtained the 2 kb upstream promoter regions of all *GhFBN* genes and analyzed their *cis*-acting regulatory elements with the PlantCARE database. In the promoter regions of *GhFBN* genes, we identified multiple types of regulatory elements (Figure 6). These elements included those responsive to plant hormones, such as ABRE (abscisic acid), CGTCA-motif and TGACG-motif (methyl jasmonate), P-box, TATC-box, and GARE-motif (gibberellic acid), AuxRR-core and TGA-element (auxin), and TCA-element (salicylic acid), indicating that the expression of *GhFBNs* is regulated by different plant hormones (Table 3). Moreover, light-responsive elements like L-box were also detected. Overall, these results suggest that *GhFBNs* participate in plant growth, development, and responses to environmental stresses.

### 3.7. Expression Profiling Based on Transcriptome Data

**Figure 7:**
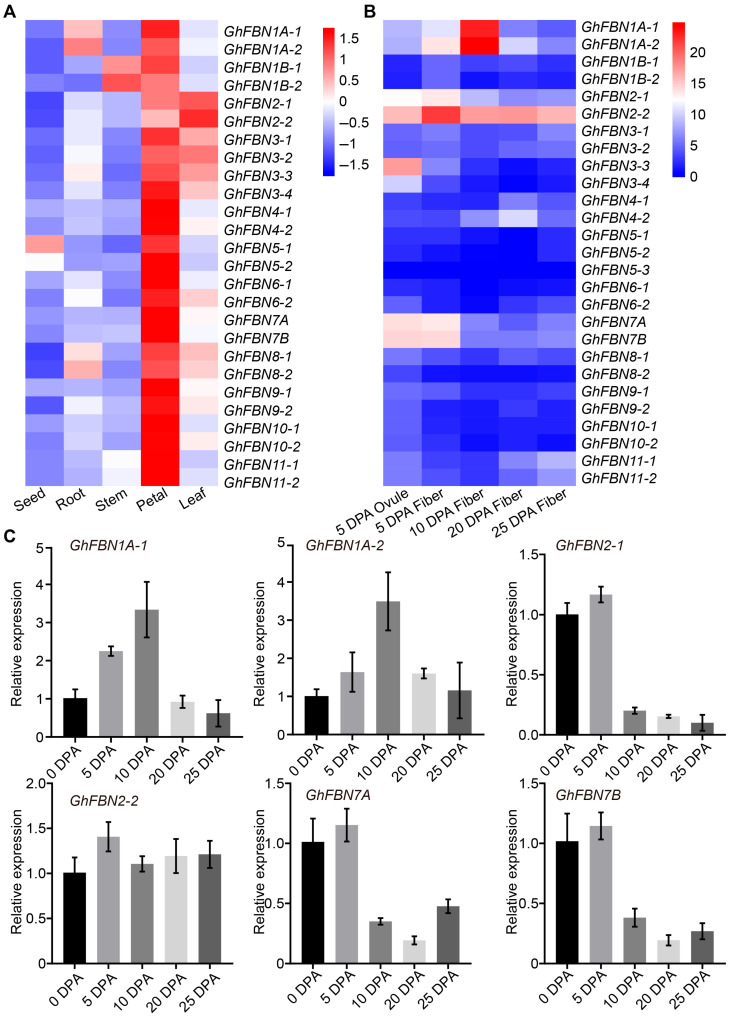
Expression analysis of *GhFBN* genes in different cotton tissues. (**A**) Expression of *GhFBNs* in seeds, roots, stems, petals and leaves. (**B**) Expression of *GhFBNs* in fibers at different developmental stages. The heatmap shows the Log2 (FPKM+1) of *GhFBNs*. Blue represents low expression, while red indicates high expression. DPA, days post-anthesis. (**C**) Expression of *GhFBN* genes in cotton fibers. Expression of *GhFBN*s is normalized based on the expression of *GhUBQ7*. Error bars represent ±SD (n = 3).

To delve deeper into the functions of *GhFBN* genes in cotton, we analyzed RNA sequencing data from various tissues of upland cotton, including seeds, roots, stems, flowers, leaves, 0 DPA ovules, and fibers at different developmental stages. The results showed that *GhFBN* genes were predominantly expressed in flowers, with leaves exhibiting the second-highest expression levels (Figure 7A). Genes including *GhFBN2-1*, *GhFBN2-2*, *GhFBN3-3*, *GhFBN7A*, and *GhFBN7B* showed elevated expression at the fiber initiation stage (Figure 7B). Significantly, *GhFBN1A-1*, *GhFBN1A-2* and *GhFBN2-2* were more highly expressed during the fiber elongation stage (Figure 7B). Notably, *GhFBN2-2* maintained consistently high expression throughout the entire fiber development process (Figure 7B). To further explore the evolutionary basis of this expression pattern, a comparative analysis of A- and D-subgenome divergence revealed distinct transcriptional dynamics among homologous genes during fiber development. Specifically, while *GhFBN2-2* (A-subgenome homoeolog) exhibits sustained high expression, its D-subgenome counterpart *GhFBN2-1* shows significantly reduced transcription during critical fiber developmental stages, suggesting minimal functional involvement in this process. In contrast, the robust, consistent expression of *GhFBN2-2* across fiber development indicates potential adaptive specialization in regulating spatiotemporal fiber developmental programs. Based on the transcriptomic expression data from different fiber development stages, we performed targeted validation of *GhFBN1A-1*, *GhFBN1A-2*, *GhFBN2-1*, *GhFBN2-2*, *GhFBN7A*, and *GhFBN7B* via quantitative real-time PCR (qRT-PCR), and the results were consistent with the expression trends observed in the transcriptomic data (Figure 7C). Collectively, these findings strongly suggest that *GhFBN*s play pivotal roles in fiber development.

**Figure 8:**
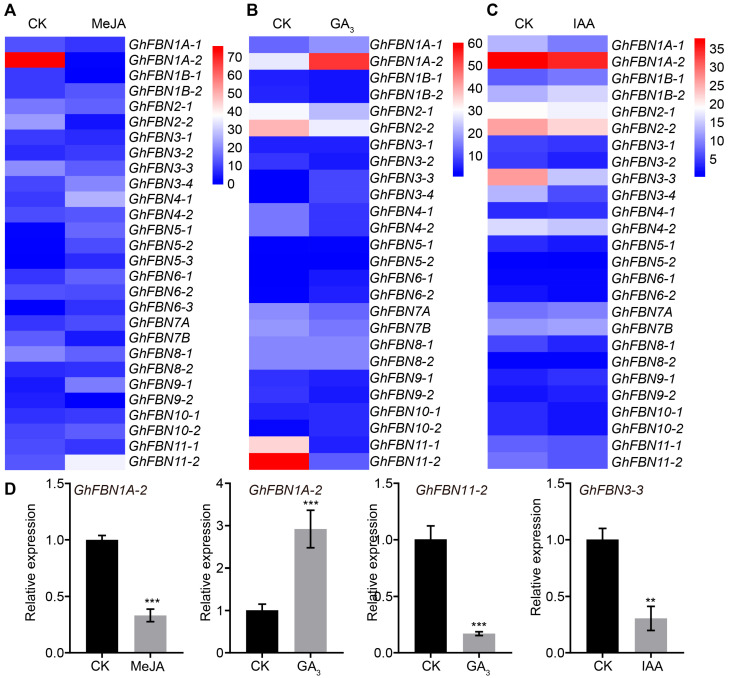
Expression profiling of *GhFBN* genes in cotton fibers at 10 DPA upon hormone treatment. (**A**) Expression of *GhFBNs* in fibers treated with MeJA. (**B**) Expression of *GhFBNs* in fibers treated with GA_3_. (**C**) Expression of *GhFBNs* in fibers treated with IAA. The heatmap shows the Log2 (FPKM+1) of *GhFBNs*. Blue represents low expression, while red indicates high expression. CK, control. MeJA, methyl jasmonate. GA_3_, gibberellin A_3_. IAA, indole-3-acetic acid. (**D**) Expression of *GhFBN* genes in cotton ovules cultured in vitro, with and without hormone treatment. Expression of *GhFBN*s is normalized based on the expression of *GhUBQ7*. Error bars represent ±SD (n = 3). Asterisks indicate significant differences by *t*-test; ** *p* ≤ 0.01; *** *p* ≤ 0.001.

Promoter analysis predicted potential involvement of *GhFBNs* in phytohormone signaling pathways. Previous studies have demonstrated that GA (gibberellin acid) and IAA can promote fiber development, whereas JA (jasmonic acid) inhibits fiber development [34,38,39]. To validate this hypothesis, transcriptomic sequencing was performed on 10 DPA fibers derived from in vitro-cultured ovules, with experimental groups treated with MeJA, GA_3_, or IAA and non-hormonally treated samples included as the control (CK). Results revealed that *GhFBN1A-2* expression was significantly downregulated by MeJA but strongly induced by GA_3_, suggesting it may have a positive regulatory role in GA_3_-mediated fiber development (Figure 8). In contrast, *GhFBN5-3* and *GhFBN6-3* are hardly expressed in fibers before and after GA_3_ and IAA treatments, indicating that they may not be involved in GA_3_- and IAA-induced fiber elongation (Figure 8B,C). Meanwhile, the expression level of *GhFBN11-2* in fiber cells was downregulated following GA_3_ treatment, while that of *GhFBN3-3* in fiber cells was similarly reduced after IAA treatment. These findings indicate that these genes exhibit a negative correlation with fiber elongation mediated by gibberellin or auxin, respectively (Figure 8B,C). Building on these transcriptomic insights, we performed qRT-PCR validation on GhFBN1A-2, GhFBN11-2, and GhFBN3-3 under identical hormonal treatments, confirming consistent expression trends with the transcriptomic data. Collectively, these data highlight the diverse regulatory roles of *GhFBNs* in phytohormone-mediated cotton fiber elongation.

## 4. Discussion

The *FBN* gene family has been identified on a whole-genome scale in numerous plant species, and considerable insights have been gained into its potential functions in the growth and development of plants such as pepper and potato [10,20,40]. Cotton, being a crucial economic crop, holds significant agricultural and industrial value. Remarkably, however, there has been no report on the whole-genome study of the *FBN* gene family in upland cotton (*Gossypium hirsutum*). Moreover, whether *FBN* genes are involved in the growth and development of cotton and, if so, what roles they play remain largely unknown, thus presenting an important area for further exploration.

From an evolutionary perspective, the number of *FBN* genes varies among different cotton species. For example, 28, 28, 13 and 15 members of the *FBN* gene family were identified in *G. hirsutum*, *G. barbadense*, *G. arboreum* and *G. raimondii*, respectively. This, together with the situations of the *FBN* gene family in other plants, indicates that the *FBN* gene family has undergone expansion and differentiation during the plant evolution process. The number of *FBN* genes in allotetraploid cotton species is approximately twice that in diploid *G. arboreum* and *G. raimondii*, suggesting a lineage-specific expansion of the *FBN* gene family during cotton polyploidization. This expansion likely provides a more abundant genetic basis for cotton to adapt to diverse environments and meet its own developmental needs.

In terms of functions, *FBN* genes play crucial roles in plant growth, development, and stress responses [41]. In cotton, different *GhFBN* genes exhibit specific expression patterns at various stages of fiber development. For instance, *GhFBN7A*, *GhFBN7B*, and other genes show high expression levels at the fiber initiation stage, while *GhFBN1A-1* and *GhFBN2-2* are more highly expressed during the fiber elongation stage. The sustained high expression of *GhFBN2-2* throughout fiber development, coupled with its distinct transcriptional divergence from the D-subgenome homoeolog *GhFBN2-1*, which shows significantly reduced transcription at critical stages, highlights subgenome-specific functional differentiation in cotton fiber traits. This expression pattern strongly supports the adaptive specialization of the A-subgenome GhFBN2-2 in regulating the spatiotemporal programs underlying fiber development, emphasizing its potential role in this agronomically vital process. This is consistent with the functions of *FBN* genes in the development processes of other plants. For example, in tomatoes, most *SlFBNs* play important roles in leaf development [22]. This indicates that *FBN* genes have conserved functions in specific tissues and developmental stages of different plants.

The differential expression of *GhFBN*s in response to MeJA, GA_3_, and IAA treatments reveals their specialized roles in hormone-mediated cotton fiber elongation. Strong induction of *GhFBN1A-2* by GA and simultaneous repression by JA highlight its potential as a positive regulator in GA-driven fiber development, while GA-induced downregulation of *GhFBN11-2* and IAA-induced downregulation of *GhFBN3-3* suggest their negative regulatory roles in hormone-induced growth. Notably, *GhFBN5-3* and *GhFBN6-3* show negligible expression changes, indicating a lack of involvement in these processes. These findings validate the involvement of *GhFBN*s in phytohormone signaling and provide critical insights for targeted molecular breeding to improve cotton fiber quality.

Regarding the response to abiotic stresses, existing studies have demonstrated that the *FBN* gene family is involved in plant responses to stresses such as drought [16,42,43]. Although there is currently no direct evidence of the specific association between *GhFBN* genes and drought stress in cotton, based on research on other plants, it can be inferred that *GhFBN* genes may also be involved. For example, in potatoes, drought stress increases the mRNA and protein abundances of CDSP 34 (FBN1) [44,45]. In wheat, the expressions of multiple *FBN* genes, such as *TaFBNA1*, are significantly upregulated under drought, low-temperature, and other stress conditions [14]. Therefore, *GhFBN* genes in cotton are likely to also play important roles in responses to drought and other abiotic stresses, which is worthy of further investigation.

In addition, the interactions between FBN proteins also affect the exertion of their functions [46]. In cotton, there is currently little research on the interactions of FBN proteins, so we refer to studies on other plants, such as the interaction between FBN1a and FBN2, and the interactions between FBN5 and SPS1, and between FBN5 and SPS2, respectively, in *Arabidopsis* [15,47]. FBN1A and FBN1B may form homodimers, heterodimers, or oligomers to interact and exert their functions [48]. It can be speculated that there is a complex interaction network among FBN proteins in cotton. These interactions may jointly regulate the growth, development, and stress response processes of cotton.

The *FBN* gene family in cotton shares similarities and specificities with those in other plants in terms of evolution, functions, and protein–protein interactions, and this study further reveals their potential in fiber quality improvement through functional characterization. Key targets are identified: leveraging *GhFBN2-2*’s consistent high expression during fiber development, enhancing *GhFBN1A-2* GA_3_-induced growth promotion, and avoiding *GhFBN11-2* and *GhFBN3-3* negative interference with hormone signaling. Future research can further focus on the specific mechanisms of cotton *FBN* genes in stress responses and their interaction relationships. This will not only contribute to a deeper exploration of the regulatory mechanisms of cotton growth and development but also provide a solid theoretical basis for the genetic improvement of cotton.

## 5. Conclusions

In conclusion, we identified 28, 28, 13 and 15 members of the *FBN* gene family in *G. hirsutum*, *G. barbadense*, *G. arboreum* and *G. raimondii*, respectively. This study provides the first comprehensive characterization of *FBN* genes in cotton and establishes the role of *GhFBN1A-2* in fiber development. By integrating analyses of gene structure, evolutionary dynamics, expression patterns, and functional validation, we delineated the evolutionary trajectories and functional diversification of *FBN* genes during cotton genome evolution. These findings not only deepen our understanding of the biological roles of *FBNs* in fiber development but also provide a foundation for future genetic improvement of cotton fiber quality. These findings deepen our understanding of *GhFBN*-mediated regulation of cotton fiber development stages like elongation and cell wall formation, provide a molecular basis for genetic improvement of fiber quality, and identify target genes to guide precision breeding for enhanced traits such as length and strength through clarified functional characteristics and regulatory patterns of specific *GhFBN* members in hormone-mediated growth.

## Figures and Tables

**Table 1:** The FBN gene family members in cotton.

Gene Name	Sequence ID	Gene (bp)	CDS (bp)	MWa (kDa)	Theoretical pI	Species
*GhFBN1A-1*	Gh_D05G1665.1	3179	978	35.42335	5.14	*G. hirsutum*
*GhFBN1A-2*	Gh_A05G1494.1	1399	978	35.44943	5.14	*G. hirsutum*
*GhFBN1B-1*	Gh_D09G0662.1	5532	1008	36.86466	4.93	*G. hirsutum*
*GhFBN1B-2*	Gh_A09G0658.1	2380	1008	36.91571	5	*G. hirsutum*
*GhFBN2-1*	Gh_D09G1905.1	4196	1107	39.83073	4.49	*G. hirsutum*
*GhFBN2-2*	Gh_A09G1782.1	2257	1101	39.62955	4.58	*G. hirsutum*
*GhFBN3-1*	Gh_A01G1493.1	3987	735	27.42963	9.65	*G. hirsutum*
*GhFBN3-2*	Gh_D01G1730.1	3179	735	27.49574	9.65	*G. hirsutum*
*GhFBN3-3*	Gh_A04G1080.1	3987	762	28.53791	9.69	*G. hirsutum*
*GhFBN3-4*	Gh_D04G1687.1	3179	762	28.50085	9.54	*G. hirsutum*
*GhFBN4-1*	Gh_D09G2214.1	4196	831	29.92041	9.37	*G. hirsutum*
*GhFBN4-2*	Gh_A09G2222.1	3179	831	30.01153	9.37	*G. hirsutum*
*GhFBN5-1*	Gh_D06G1644.1	5532	1302	47.95253	6	*G. hirsutum*
*GhFBN5-2*	Gh_A06G1315.1	1399	1500	55.18532	9	*G. hirsutum*
*GhFBN5-3*	Gh_D11G3441.1	4196	429	15.74633	8.67	*G. hirsutum*
*GhFBN6-1*	Gh_D06G1142.1	5532	789	28.93921	9.4	*G. hirsutum*
*GhFBN6-2*	Gh_A06G0966.1	1399	789	28.98224	9.4	*G. hirsutum*
*GhFBN6-3*	Gh_D01G0744.1	3179	348	12.79046	8.06	*G. hirsutum*
*GhFBN7A*	Gh_A09G1197.1	2380	912	33.32015	6.67	*G. hirsutum*
*GhFBN7B*	Gh_D09G1203.1	4196	912	33.29012	6.67	*G. hirsutum*
*GhFBN8-1*	Gh_D11G1079.1	4196	714	26.46673	8.78	*G. hirsutum*
*GhFBN8-2*	Gh_A11G0937.1	3179	846	31.60553	9.22	*G. hirsutum*
*GhFBN9-1*	Gh_D07G1476.1	5532	651	24.49891	8.61	*G. hirsutum*
*GhFBN9-2*	Gh_A07G1372.1	1399	645	24.35177	8.61	*G. hirsutum*
*GhFBN10-1*	Gh_D08G0826.1	5532	1200	44.77003	6.48	*G. hirsutum*
*GhFBN10-2*	Gh_A08G0709.1	1399	1200	44.78029	8.64	*G. hirsutum*
*GhFBN11-1*	Gh_D12G2421.1	4196	2031	76.64194	9.74	*G. hirsutum*
*GhFBN11-2*	Gh_A12G2271.1	3179	2031	76.67501	9.86	*G. hirsutum*
*GaFBN1A*	Ga05G1857.1	1399	978	35.43737	5.14	*G. arboreum*
*GaFBN1B*	Ga09G0772.1	2379	1008	36.92974	5	*G. arboreum*
*GaFBN2*	Ga09G2235.1	1607	1101	39.6435	4.54	*G. arboreum*
*GaFBN3A-1*	Ga02G1224.1	2259	735	27.44765	9.65	*G. arboreum*
*GaFBN3A-2*	Ga04G0211.1	1736	762	28.54992	9.69	*G. arboreum*
*GaFBN4*	Ga09G2612.1	1590	831	29.95748	9.37	*G. arboreum*
*GaFBN5*	Ga11G1531.1	2064	867	32.03204	7.55	*G. arboreum*
*GaFBN6*	Ga06G1253.1	1113	705	25.7124	9.08	*G. arboreum*
*GaFBN7A*	Ga09G1460.1	5543	912	33.29209	6.07	*G. arboreum*
*GaFBN8*	Ga11G2969.1	3024	846	31.58558	9.07	*G. arboreum*
*GaFBN9*	Ga07G1647.1	1031	645	24.35177	8.61	*G. arboreum*
*GaFBN10*	Ga08G0915.1	5511	1200	44.73919	8.65	*G. arboreum*
*GaFBN11*	Ga12G0290.1	4018	2031	76.67501	9.86	*G. arboreum*
*GbFBN1A-1*	Gbar_D05G017830.1	2891	978	35.40735	5.14	*G. barbadense*
*GbFBN1A-2*	Gbar_A05G017410.1	2838	978	35.44943	5.14	*G. barbadense*
*GbFBN1A-3*	Gbar_D09G007520.1	2745	1008	36.87478	4.99	*G. barbadense*
*GbFBN1B*	Gbar_A09G007800.1	2780	1008	36.90267	4.95	*G. barbadense*
*GbFBN2-1*	Gbar_D09G020830.1	2524	1107	39.81765	4.49	*G. barbadense*
*GbFBN2-2*	Gbar_A09G021110.1	2508	1101	39.65757	4.58	*G. barbadense*
*GbFBN3A-1*	Gbar_D01G018650.1	5258	747	28.08431	9.34	*G. barbadense*
*GbFBN3A-2*	Gbar_A01G017480.1	2517	735	27.42963	9.65	*G. barbadense*
*GbFBN3A-4*	Gbar_A04G013910.1	1706	762	28.53392	9.69	*G. barbadense*
*GbFBN3A-3*	Gbar_D04G018570.1	1864	762	28.49887	9.54	*G. barbadense*
*GbFBN4-1*	Gbar_D09G024190.1	2000	831	29.91642	9.37	*G. barbadense*
*GbFBN4-2*	Gbar_A09G024530.1	1568	813	29.46222	8.77	*G. barbadense*
*GbFBN5-1*	Gbar_D06G016800.1	3442	1614	59.91259	7.03	*G. barbadense*
*GbFBN5-2*	Gbar_A06G016120.1	2029	897	33.38748	7.64	*G. barbadense*
*GbFBN5-3*	Gbar_D11G024180.1	12587	894	33.03026	9.27	*G. barbadense*
*GbFBN5-4*	Gbar_A11G023440.1	2514	552	20.34894	9.43	*G. barbadense*
*GbFBN6-1*	Gbar_D06G011790.1	1462	705	25.69841	9.19	*G. barbadense*
*GbFBN6-2*	Gbar_A06G011340.1	1453	717	26.18494	8.94	*G. barbadense*
*GbFBN7B-1*	Gbar_D09G013520.1	6058	912	33.29012	6.67	*G. barbadense*
*GbFBN7A-2*	Gbar_A09G013680.1	5971	912	33.32015	6.67	*G. barbadense*
*GbFBN8-1*	Gbar_D11G011270.1	3392	846	31.58953	9.25	*G. barbadense*
*GbFBN8-2*	Gbar_A11G010770.1	3987	1014	37.98029	9.67	*G. barbadense*
*GbFBN9-1*	Gbar_D07G016160.1	3560	651	24.49891	8.61	*G. barbadense*
*GbFBN9-2*	Gbar_A07G015770.1	3386	645	24.35177	8.61	*G. barbadense*
*GbFBN10-1*	Gbar_D08G008730.1	5745	954	35.6023	6.46	*G. barbadense*
*GbFBN10-2*	Gbar_A08G008420.1	5752	1119	41.6738	7.7	*G. barbadense*
*GbFBN11-1*	Gbar_D12G026080.1	4751	1425	54.09186	9.87	*G. barbadense*
*GbFBN11-2*	Gbar_A12G026140.1	4788	2031	76.68903	9.86	*G. barbadense*
*GrFBN1A-1*	Gorai.009G182500.1	2513	978	35.42335	5.14	*G. raimondii*
*GrFBN1A-2*	Gorai.006G082400.1	2754	1014	37.13302	5.06	*G. raimondii*
*GrFBN2*	Gorai.006G217700.1	2488	1125	40.3022	4.49	*G. raimondii*
*GrFBN3A-1*	Gorai.002G209300.1	2559	735	27.42461	9.56	*G. raimondii*
*GrFBN3A-2*	Gorai.012G160700.1	1982	762	28.48682	9.54	*G. raimondii*
*GrFBN4*	Gorai.006G252600.1	2015	831	29.97849	9.37	*G. raimondii*
*GrFBN5-1*	Gorai.010G181700.1	3037	1812	67.68879	8.5	*G. raimondii*
*GrFBN5-2*	Gorai.002G151300.1	1994	813	30.16	9.12	*G. raimondii*
*GrFBN6-1*	Gorai.010G123700.1	1498	705	25.68439	9.19	*G. raimondii*
*GrFBN6-2*	Gorai.002G101300.1	728	423	15.86637	9.16	*G. raimondii*
*GrFBN7B*	Gorai.006G142800.1	6103	912	33.29012	6.67	*G. raimondii*
*GrFBN8*	Gorai.007G115100.1	3493	861	32.16422	9.36	*G. raimondii*
*GrFBN9*	Gorai.001G175700.1	3596	651	24.5059	8.61	*G. raimondii*
*GrFBN10*	Gorai.004G092800.1	5850	1200	44.80911	6.93	*G. raimondii*
*GrFBN11*	Gorai.008G269500.1	4726	2031	76.79095	9.76	*G. raimondii*

**Table 2:** Ka/Ks analysis of *GhFBN* gene families in *Gossypium hirsutum*.

Sequence ID	Ka	Ks	Ka/Ks
*GhFBN3-1&GhFBN3-3*	0.25646808027089535	1.8899984771994518	0.1356975063021864
*GhFBN3-1&GhFBN3-2*	0.015378195927205629	0.031811997515935844	0.48340868628265493
*GhFBN3-1&GhFBN3-4*	0.26519839929406197	2.0183731848080146	0.1313921534878533
*GhFBN3-3&GhFBN3-2*	0.2539649643874308	1.9922119769228535	0.12747888644847022
*GhFBN3-3&GhFBN3-4*	0.010415327224946307	0.022316848711233652	0.4667024166231648
*GhFBN1A-2&GhFBN1B-2*	0.11398702336283616	0.6464385028816231	0.17633080773301285
*GhFBN1A-2&GhFBN1A-1*	0.002682766106657679	0.026415055967744308	0.10156200728605806
*GhFBN1A-2&GhFBN1B-1*	0.11237961274006769	0.6372917984117465	0.17633933626658815
*GhFBN6-2&GhFBN6-1*	0.013447621783443438	0.03803440541457851	0.35356466433123185
*GhFBN5-2&GhFBN5-1*	0.037268406863882296	0.05082235814271873	0.7333073124868705
*GhFBN9-2&GhFBN9-1*	0.006157038170299509	0.0812977842242655	0.07573439090682837
*GhFBN10-2&GhFBN10-1*	0.014196438052508897	0.025840759627618143	0.5493816070846461
*GhFBN1B-2&GhFBN1A-1*	0.11091080011024723	0.6246818422013267	0.17754766125329788
*GhFBN1B-2&GhFBN1B-1*	0.014403061291802292	0.0434431959190317	0.33153779290654267
*GhFBN7A&GhFBN7B*	0.004380640343959717	0.04578080007805861	0.0956872823648888
*GhFBN2-2&GhFBN2-1*	0.009753261495566346	0.04104225646267837	0.23763950465139363
*GhFBN8-2&GhFBN8-1*	0.03976139397807425	0.04769566096457199	0.8336480336777121
*GhFBN11-2&GhFBN11-1*	0.008441190776946137	0.02530551062085989	0.3335712487061169
*GhFBN3-2&GhFBN3-4*	0.2626554249946424	2.1422101503898068	0.12260955114363936
*GhFBN1A-1&GhFBN1B-1*	0.11243405990473135	0.6358361961198246	0.17682865585642585

**Table 3:** Analysis of cis-acting elements in *GhFBN*.

ID	Cis-Elements Name	Functions of Cis-Elements
Gh_A01G1493	ABRE	cis-acting element involved in abscisic acid responsiveness
Gh_A01G1493	ABRE	cis-acting element involved in abscisic acid responsiveness
Gh_A04G1080	CGTCA-motif	cis-acting regulatory element involved in MeJA responsiveness
Gh_A04G1080	TATC-box	cis-acting element involved in gibberellin responsiveness
Gh_A04G1080	TGACG-motif	cis-acting regulatory element involved in MeJA responsiveness
Gh_A04G1080	ABRE	cis-acting element involved in abscisic acid responsiveness
Gh_A04G1080	ABRE	cis-acting element involved in abscisic acid responsiveness
Gh_A04G1080	ABRE	cis-acting element involved in abscisic acid responsiveness
Gh_A04G1080	AuxRR-core	cis-acting regulatory element involved in auxin responsiveness
Gh_A05G1494	P-box	gibberellin-responsive element
Gh_A05G1494	CGTCA-motif	cis-acting regulatory element involved in MeJA responsiveness
Gh_A05G1494	ABRE	cis-acting element involved in abscisic acid responsiveness
Gh_A05G1494	ABRE	cis-acting element involved in abscisic acid responsiveness
Gh_A05G1494	ABRE	cis-acting element involved in abscisic acid responsiveness
Gh_A05G1494	ABRE	cis-acting element involved in abscisic acid responsiveness
Gh_A05G1494	ABRE	cis-acting element involved in abscisic acid responsiveness
Gh_A05G1494	TGACG-motif	cis-acting regulatory element involved in MeJA responsiveness
Gh_A05G1494	TCA-element	cis-acting element involved in salicylic acid responsiveness
Gh_A05G1494	GARE-motif	gibberellin-responsive element
Gh_A06G0966	CGTCA-motif	cis-acting regulatory element involved in MeJA responsiveness
Gh_A06G0966	TATC-box	cis-acting element involved in gibberellin responsiveness
Gh_A06G0966	ABRE	cis-acting element involved in abscisic acid responsiveness
Gh_A06G0966	ABRE	cis-acting element involved in abscisic acid responsiveness
Gh_A06G0966	ABRE	cis-acting element involved in abscisic acid responsiveness
Gh_A06G0966	ABRE	cis-acting element involved in abscisic acid responsiveness
Gh_A06G0966	TGACG-motif	cis-acting regulatory element involved in MeJA responsiveness
Gh_A06G1315	TCA-element	cis-acting element involved in salicylic acid responsiveness
Gh_A06G1315	AuxRR-core	cis-acting regulatory element involved in auxin responsiveness
Gh_A06G1315	ABRE	cis-acting element involved in abscisic acid responsiveness
Gh_A07G1372	ABRE	cis-acting element involved in abscisic acid responsiveness
Gh_A07G1372	ABRE	cis-acting element involved in abscisic acid responsiveness
Gh_A07G1372	TATC-box	cis-acting element involved in gibberellin responsiveness
Gh_A08G0709	GARE-motif	gibberellin-responsive element
Gh_A09G0658	CGTCA-motif	cis-acting regulatory element involved in MeJA responsiveness
Gh_A09G0658	TGACG-motif	cis-acting regulatory element involved in MeJA responsiveness
Gh_A09G0658	TCA-element	cis-acting element involved in salicylic acid responsiveness
Gh_A09G1197	TATC-box	cis-acting element involved in gibberellin responsiveness
Gh_A09G1197	TCA-element	cis-acting element involved in salicylic acid responsiveness
Gh_A09G1782	ABRE	cis-acting element involved in abscisic acid responsiveness
Gh_A09G1782	AuxRR-core	cis-acting regulatory element involved in auxin responsiveness
Gh_A09G1782	TGACG-motif	cis-acting regulatory element involved in MeJA responsiveness
Gh_A09G1782	CGTCA-motif	cis-acting regulatory element involved in MeJA responsiveness
Gh_A09G2222	ABRE	cis-acting element involved in abscisic acid responsiveness
Gh_A09G2222	ABRE	cis-acting element involved in abscisic acid responsiveness
Gh_A09G2222	TGACG-motif	cis-acting regulatory element involved in MeJA responsiveness
Gh_A09G2222	TCA-element	cis-acting element involved in salicylic acid responsiveness
Gh_A09G2222	P-box	gibberellin-responsive element
Gh_A09G2222	CGTCA-motif	cis-acting regulatory element involved in MeJA responsiveness
Gh_A11G0937	GARE-motif	gibberellin-responsive element
Gh_A11G0937	TGACG-motif	cis-acting regulatory element involved in MeJA responsiveness
Gh_A11G0937	CGTCA-motif	cis-acting regulatory element involved in MeJA responsiveness
Gh_A11G0937	TGA-element	auxin-responsive element
Gh_A11G0937	TATC-box	cis-acting element involved in gibberellin responsiveness
Gh_A11G0937	TATC-box	cis-acting element involved in gibberellin responsiveness
Gh_A11G0937	P-box	gibberellin-responsive element
Gh_A12G2271	TCA-element	cis-acting element involved in salicylic acid responsiveness
Gh_A12G2271	GARE-motif	gibberellin-responsive element
Gh_A12G2271	ABRE	cis-acting element involved in abscisic acid responsiveness
Gh_A12G2271	ABRE	cis-acting element involved in abscisic acid responsiveness
Gh_A12G2271	ABRE	cis-acting element involved in abscisic acid responsiveness
Gh_A12G2271	ABRE	cis-acting element involved in abscisic acid responsiveness
Gh_A12G2271	ABRE	cis-acting element involved in abscisic acid responsiveness
Gh_A12G2271	ABRE	cis-acting element involved in abscisic acid responsiveness
Gh_A12G2271	ABRE	cis-acting element involved in abscisic acid responsiveness
Gh_A12G2271	ABRE	cis-acting element involved in abscisic acid responsiveness
Gh_A12G2271	TGACG-motif	cis-acting regulatory element involved in MeJA responsiveness
Gh_A12G2271	TGACG-motif	cis-acting regulatory element involved in MeJA responsiveness
Gh_A12G2271	TGACG-motif	cis-acting regulatory element involved in MeJA responsiveness
Gh_A12G2271	CGTCA-motif	cis-acting regulatory element involved in MeJA responsiveness
Gh_A12G2271	CGTCA-motif	cis-acting regulatory element involved in MeJA responsiveness
Gh_A12G2271	CGTCA-motif	cis-acting regulatory element involved in MeJA responsiveness
Gh_D01G0744	TGA-element	auxin-responsive element
Gh_D01G0744	P-box	gibberellin-responsive element
Gh_D01G0744	GARE-motif	gibberellin-responsive element
Gh_D01G0744	TCA-element	cis-acting element involved in salicylic acid responsiveness
Gh_D01G0744	TCA-element	cis-acting element involved in salicylic acid responsiveness
Gh_D01G0744	ABRE	cis-acting element involved in abscisic acid responsiveness
Gh_D01G1730	ABRE	cis-acting element involved in abscisic acid responsiveness
Gh_D01G1730	ABRE	cis-acting element involved in abscisic acid responsiveness
Gh_D01G1730	AuxRR-core	cis-acting regulatory element involved in auxin responsiveness
Gh_D04G1687	ABRE	cis-acting element involved in abscisic acid responsiveness
Gh_D04G1687	ABRE	cis-acting element involved in abscisic acid responsiveness
Gh_D04G1687	AuxRR-core	cis-acting regulatory element involved in auxin responsiveness
Gh_D04G1687	TGACG-motif	cis-acting regulatory element involved in MeJA responsiveness
Gh_D04G1687	CGTCA-motif	cis-acting regulatory element involved in MeJA responsiveness
Gh_D04G1687	P-box	gibberellin-responsive element
Gh_D04G1687	TATC-box	cis-acting element involved in gibberellin responsiveness
Gh_D05G1665	TCA-element	cis-acting element involved in salicylic acid responsiveness
Gh_D05G1665	TCA-element	cis-acting element involved in salicylic acid responsiveness
Gh_D05G1665	GARE-motif	gibberellin-responsive element
Gh_D05G1665	ABRE	cis-acting element involved in abscisic acid responsiveness
Gh_D05G1665	ABRE	cis-acting element involved in abscisic acid responsiveness
Gh_D05G1665	ABRE	cis-acting element involved in abscisic acid responsiveness
Gh_D05G1665	ABRE	cis-acting element involved in abscisic acid responsiveness
Gh_D05G1665	ABRE	cis-acting element involved in abscisic acid responsiveness
Gh_D05G1665	TGACG-motif	cis-acting regulatory element involved in MeJA responsiveness
Gh_D05G1665	CGTCA-motif	cis-acting regulatory element involved in MeJA responsiveness
Gh_D05G1665	P-box	gibberellin-responsive element
Gh_D05G1665	TATC-box	cis-acting element involved in gibberellin responsiveness
Gh_D06G1142	TGACG-motif	cis-acting regulatory element involved in MeJA responsiveness
Gh_D06G1142	AuxRR-core	cis-acting regulatory element involved in auxin responsiveness
Gh_D06G1142	ABRE	cis-acting element involved in abscisic acid responsiveness
Gh_D06G1142	ABRE	cis-acting element involved in abscisic acid responsiveness
Gh_D06G1142	ABRE	cis-acting element involved in abscisic acid responsiveness
Gh_D06G1142	ABRE	cis-acting element involved in abscisic acid responsiveness
Gh_D06G1142	ABRE	cis-acting element involved in abscisic acid responsiveness
Gh_D06G1142	TATC-box	cis-acting element involved in gibberellin responsiveness
Gh_D06G1142	CGTCA-motif	cis-acting regulatory element involved in MeJA responsiveness
Gh_D06G1644	P-box	gibberellin-responsive element
Gh_D06G1644	CGTCA-motif	cis-acting regulatory element involved in MeJA responsiveness
Gh_D06G1644	TGA-element	auxin-responsive element
Gh_D06G1644	ABRE	cis-acting element involved in abscisic acid responsiveness
Gh_D06G1644	ABRE	cis-acting element involved in abscisic acid responsiveness
Gh_D06G1644	TGACG-motif	cis-acting regulatory element involved in MeJA responsiveness
Gh_D06G1644	TCA-element	cis-acting element involved in salicylic acid responsiveness
Gh_D07G1476	ABRE	cis-acting element involved in abscisic acid responsiveness
Gh_D07G1476	ABRE	cis-acting element involved in abscisic acid responsiveness
Gh_D07G1476	GARE-motif	gibberellin-responsive element
Gh_D07G1476	TGA-element	auxin-responsive element
Gh_D08G0826	ABRE	cis-acting element involved in abscisic acid responsiveness
Gh_D08G0826	ABRE	cis-acting element involved in abscisic acid responsiveness
Gh_D08G0826	ABRE	cis-acting element involved in abscisic acid responsiveness
Gh_D09G0662	ABRE	cis-acting element involved in abscisic acid responsiveness
Gh_D09G0662	TCA-element	cis-acting element involved in salicylic acid responsiveness
Gh_D09G1203	P-box	gibberellin-responsive element
Gh_D09G1203	CGTCA-motif	cis-acting regulatory element involved in MeJA responsiveness
Gh_D09G1203	ABRE	cis-acting element involved in abscisic acid responsiveness
Gh_D09G1203	ABRE	cis-acting element involved in abscisic acid responsiveness
Gh_D09G1203	TGACG-motif	cis-acting regulatory element involved in MeJA responsiveness
Gh_D09G1203	TCA-element	cis-acting element involved in salicylic acid responsiveness
Gh_D09G2214	ABRE	cis-acting element involved in abscisic acid responsiveness
Gh_D09G2214	ABRE	cis-acting element involved in abscisic acid responsiveness
Gh_D09G2214	ABRE	cis-acting element involved in abscisic acid responsiveness
Gh_D09G2214	ABRE	cis-acting element involved in abscisic acid responsiveness
Gh_D09G2214	ABRE	cis-acting element involved in abscisic acid responsiveness
Gh_D09G2214	ABRE	cis-acting element involved in abscisic acid responsiveness
Gh_D09G2214	TGACG-motif	cis-acting regulatory element involved in MeJA responsiveness
Gh_D09G2214	TGACG-motif	cis-acting regulatory element involved in MeJA responsiveness
Gh_D09G2214	TGACG-motif	cis-acting regulatory element involved in MeJA responsiveness
Gh_D09G2214	CGTCA-motif	cis-acting regulatory element involved in MeJA responsiveness
Gh_D09G2214	CGTCA-motif	cis-acting regulatory element involved in MeJA responsiveness
Gh_D09G2214	CGTCA-motif	cis-acting regulatory element involved in MeJA responsiveness
Gh_D11G1079	P-box	gibberellin-responsive element
Gh_D11G1079	TATC-box	cis-acting element involved in gibberellin responsiveness
Gh_D11G1079	TATC-box	cis-acting element involved in gibberellin responsiveness
Gh_D11G1079	TGA-element	auxin-responsive element
Gh_D11G1079	ABRE	cis-acting element involved in abscisic acid responsiveness
Gh_D11G1079	GARE-motif	gibberellin-responsive element
Gh_D11G3441	CGTCA-motif	cis-acting regulatory element involved in MeJA responsiveness
Gh_D11G3441	CGTCA-motif	cis-acting regulatory element involved in MeJA responsiveness
Gh_D11G3441	TGACG-motif	cis-acting regulatory element involved in MeJA responsiveness
Gh_D11G3441	TGACG-motif	cis-acting regulatory element involved in MeJA responsiveness
Gh_D12G2421	CGTCA-motif	cis-acting regulatory element involved in MeJA responsiveness
Gh_D12G2421	CGTCA-motif	cis-acting regulatory element involved in MeJA responsiveness
Gh_D12G2421	GARE-motif	gibberellin-responsive element
Gh_D12G2421	TCA-element	cis-acting element involved in salicylic acid responsiveness
Gh_D12G2421	TGACG-motif	cis-acting regulatory element involved in MeJA responsiveness
Gh_D12G2421	TGACG-motif	cis-acting regulatory element involved in MeJA responsiveness
Gh_D12G2421	ABRE	cis-acting element involved in abscisic acid responsiveness
Gh_D12G2421	ABRE	cis-acting element involved in abscisic acid responsiveness
Gh_D12G2421	ABRE	cis-acting element involved in abscisic acid responsiveness
Gh_D12G2421	ABRE	cis-acting element involved in abscisic acid responsiveness
Gh_D12G2421	ABRE	cis-acting element involved in abscisic acid responsiveness
Gh_D12G2421	ABRE	cis-acting element involved in abscisic acid responsiveness
Gh_D12G2421	ABRE	cis-acting element involved in abscisic acid responsiveness
Gh_D09G1905	L-box	part of a light-responsive element

## Data Availability

The data that supports this study are available in the Supplementary Materials of this article.

## Supplementary Materials

The following supporting information can be downloaded at https://www.mdpi.com/article/10.3390/biology14081012/s1. Table S1: The primers used in this study.
